# Effects of plant-based diet on metabolic parameters, liver and kidney steatosis: a prospective interventional open-label study

**DOI:** 10.1017/S0007114525000017

**Published:** 2025-02-14

**Authors:** Begum Guler Senturk, Bengi Gurses, Ceren Soyturk, Sidar Copur, Said Incir, Dimitrie Siriopol, Nuri Baris Hasbal, Murat Akyildiz, Daniel H van Raalte, Mehmet Kanbay

**Affiliations:** 1 Department of Internal Medicine, Koc University School of Medicine, Istanbul, Turkey; 2 Department of Radiology, Koc University School of Medicine, Istanbul, Turkey; 3 Clinical Dietician, Koc University Hospital, Istanbul, Turkey; 4 Department of Clinical Biochemistry, Koc University School of Medicine, Istanbul, Turkey; 5 Department of Nephrology, ‘Saint John the New’ County Hospital, Suceava, Romania; 6 Division of Nephrology, Department of Internal Medicine, Koc University School of Medicine, Istanbul, Turkey; 7 Division of Gastroenterology, Department of Internal Medicine, Koc University School of Medicine, Istanbul, Turkey; 8 Diabetes Center, Department of Internal Medicine, Amsterdam University Medical Centers, Amsterdam, The Netherlands

**Keywords:** Vegetarian diet, Non-alcoholic fatty liver disease, Chronic kidney disease, Steatosis, Hepatosteatosis

## Abstract

This interventional single-centre prospective open-label study aims to evaluate the effects of a vegan diet, compared with a vegetarian and omnivorous diet, on metabolic parameters, insulin sensitivity, and liver and kidney steatosis in healthy adults. The study included fifty-three omnivorous participants aged 18–40 years, BMI 18–30 kg/m^2^, without any chronic disease, chronic medication use, active smoking or significant alcohol consumption. All participants were omnivorous at baseline and selected to continue an omnivorous diet or transition to a vegetarian or vegan diet, with follow-up over 6 months. Anthropometric measurements, biochemical parameters and liver and kidney steatosis were assessed at baseline and after six months using MRI-proton density fat fraction. Primary outcomes included changes in liver and kidney steatosis, while secondary outcomes were alterations in anthropometric and biochemical markers. Among fifty-three participants, eighteen followed an omnivorous diet, twenty-one adopted a vegetarian diet and fourteen transitioned to a vegan diet. Dietary interventions did not result in statistically significant changes in BMI, fat mass, fat percentage or muscle mass over 6 months. However, statistically significant improvements in systolic and diastolic blood pressure, favouring the vegan diet, were observed. We aimed to control for potentially confounding variables to ensure the reliability of these findings. We have demonstrated a better decline in steatosis at the lower kidney pole, the total hilus and the Liver 6 index in vegans. We demonstrated that a plant-based diet is associated with improvements in several metabolic parameters and may reduce liver and kidney steatosis.

Non-alcoholic fatty liver disease (NAFLD) is the most common cause of chronic liver disease globally. It has an estimated prevalence of 25·2 %^(1)^. Common risk factors include high fructose intake, diabetes mellitus, metabolic syndrome, hyperlipidemia, a Western-type diet, obesity, polycystic ovary syndrome and obstructive sleep apnoea^(1,2)^. Recently, NAFLD has been considered a component of the metabolic syndrome and referred to as metabolic dysfunction-associated fatty liver disease, with a range from simple steatosis to non-alcoholic steatosis^(3)^. The most widely accepted pathophysiological explanation is the ‘two-hit hypothesis’. The first hit involves lipid accumulation in the liver, primarily mediated by peripheral insulin resistance. The second hit is characterised by a pro-inflammatory, pro-oxidant and pro-fibrotic immune response to this accumulation^(2,4–6)^. Since there are no approved pharmacotherapies available for the treatment of NAFLD, dietary and lifestyle modifications are the mainstay of preventive and therapeutic approaches for patients with or at risk of NAFLD^(7,8)^. Despite growing concern about the pathophysiological and therapeutic roles of dietary habits in NAFLD and insulin resistance, there is no consensus regarding the recommended dietary routine. A systematic review of forty-eight studies, including twelve cohort studies and thirty-six cross-sectional studies, reported that vegan diets are lower in protein intake and certain micronutrients (i.e. vitamin B_12_, riboflavin, niacin, Ca, Se, Zn and iodine) compared with omnivorous diets, though these intakes generally meet daily requirements^(9,10)^. Large-scale clinical studies have demonstrated that vegetarian or vegan diets are associated with considerable protection against CVD, metabolic syndrome, insulin resistance and chronic kidney disease^(11–14)^. Multiple randomised controlled trials have also shown that vegan diets positively affect body fat composition and insulin sensitivity^(15–17)^. Patients with chronic kidney disease who incorporated plant-based proteins into their diet demonstrated a reduced incidence of disease progression and mortality^(18,19)^. A recent study revealed that lacto-ovo-vegetarian diet leads to improvements in liver health markers, such as reductions in liver enzymes, in individuals with NAFLD^(20)^. With this background in mind, in this prospective study, we aimed to evaluate the effects of plant-based diets, specifically vegetarian and vegan diets, compared with omnivore diets on multiple variables, including the steatosis of the liver and kidneys, biochemical parameters including serum lipid profile, liver enzymes, and insulin resistance, and clinical parameters such as blood pressure (BP), BMI, and body fat or muscle mass.

## Materials and methods

We have designed an interventional open-label prospective study investigating the effects of three different diets – omnivorous, vegetarian and vegan – on multiple health outcomes over a 6-month period (October 2021–July 2022). Both the intervention and follow-up were conducted during this 6-month time frame. This study adhered to the guidelines of the Declaration of Helsinki, and all procedures involving human subjects were approved by the Koç University School of Medicine (2021.216.IRB1.073). Written informed consent was obtained from all patients. Trial registration number: NCT05351853.

### Participants selection

Participants were recruited through an online application form, which was advertised via social media platforms such as X (formerly known as Twitter), Instagram and institutional announcements. The form included detailed information about the study’s aim, design, follow-up periods and the primary and secondary outcomes being investigated. Applicants were then contacted via telephone interviews, during which further details about the study procedures were provided. The inclusion criteria included participants aged 18–40 years with a BMI of 18–30 kg/m^2^ who were omnivorous at baseline. The exclusion criteria included a history of chronic systemic diseases (such as CVD, diabetes, chronic kidney disease, chronic respiratory disease, inflammatory bowel disease, liver disease and autoimmune disorders like rheumatoid arthritis), chronic medication use, active smoking and alcohol consumption exceeding 10 g/d for females and 20 g/d for males. Participants following a vegan or vegetarian diet at baseline were also excluded.

Participants were assigned to the omnivorous, vegetarian or vegan diet groups based on their preferences, as randomisation was not feasible due to the challenges of maintaining adherence to specific dietary interventions. All participants were omnivorous at baseline and were given the choice to either continue with their omnivorous diet or transition to a vegetarian or vegan diet, according to their willingness and interest. This self-selection approach allowed participants to align their choice with personal dietary preferences, which may have supported adherence throughout the study period. To control for potential baseline differences, participants were matched by age, gender and BMI. Informed consent was obtained from all participants.

**Fig. 1.**
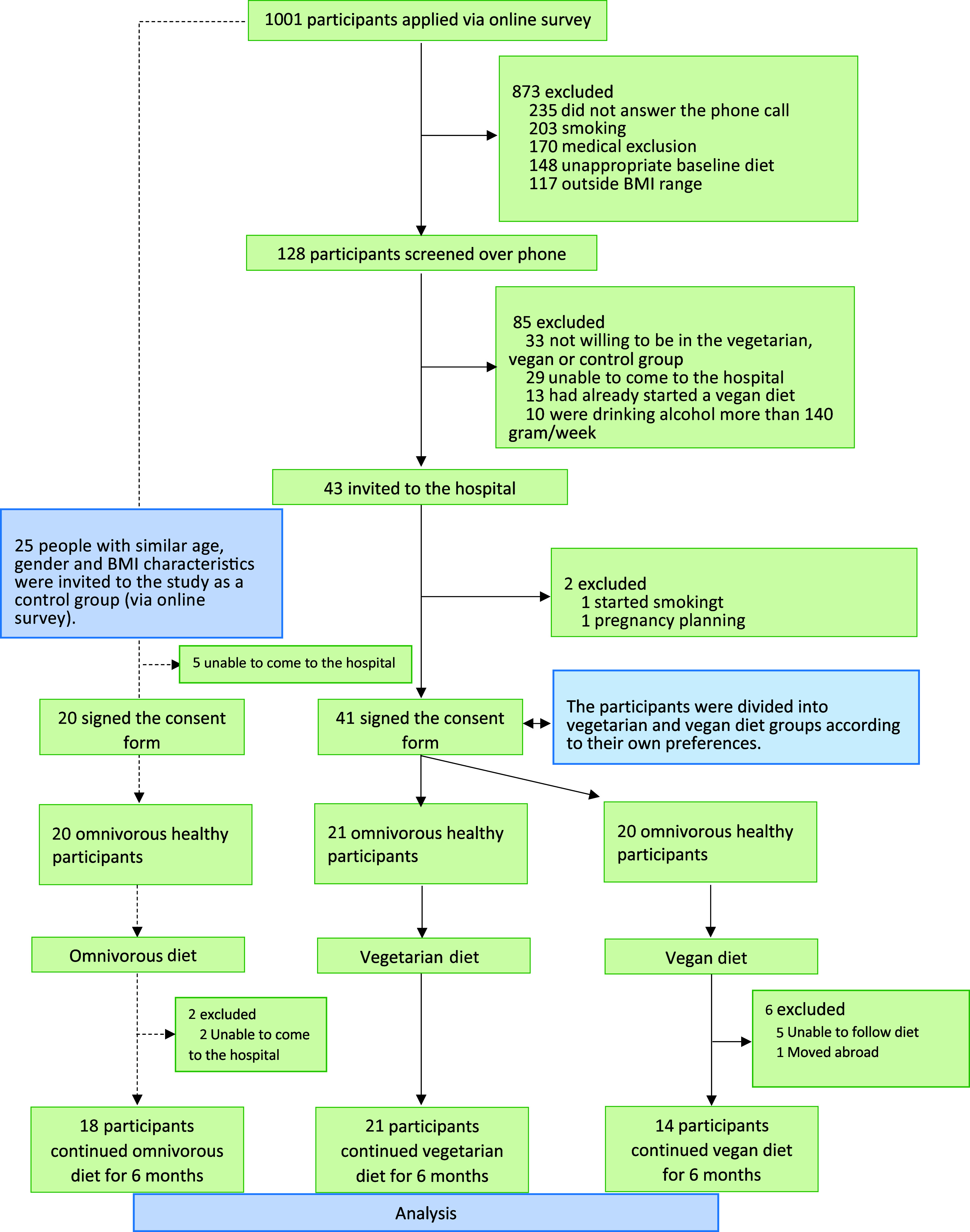
Study design.

The minimum sample size required to conduct this interventional study was calculated to be 31, with a CI of 95 % and a prevalence of veganism of 2 % in the general population^(21,22)^. The study flow chart is presented in Fig. 1.

### Study design and investigated parameters

Participants were divided into three groups based on their dietary preferences: (1) those who continued their omnivorous diet, (2) those who switched to a vegetarian diet and (3) those who switched to a vegan diet. All participants were counselled by the same nutritional specialist at baseline and monthly thereafter. Counselling sessions included guidance on dietary patterns, daily energy needs (calculated via the Schofield formula)^(23)^, daily exercise routines, macro- and micronutrients and diet. While no specific food or beverage restrictions were imposed, participants were expected to adhere to their assigned dietary group. There was no upper limit on daily energetic intake as long as the prescribed diet was followed.

Participants’ adherence to their assigned diet was systematically monitored each month through qualitative assessments (in-depth interviews) and quantitative assessments (mean daily energetic intake), based on 3-d food logs kept by participants at monthly intervals. A registered dietitian met with each participant monthly, during which 3-d food logs were completed at each session, resulting in six logs over the study period. These logs were used to calculate compliance with the prescribed diet, allowing adherence levels to be quantified for each dietary group. All groups demonstrated high compliance with their assigned diets throughout the study, as observed through monthly food logs and qualitative assessments conducted by the dietitian. Additionally, participants’ physical activity was tracked using a mobile phone application that recorded daily step counts. Counselling sessions included general recommendations for physical activity, which participants were encouraged to follow. Baseline physical activity levels are reported in Table 1, and changes in activity were monitored and assessed at both baseline and the 6-month follow-up to capture any shifts over the study period.

**Table 1.** Baseline characteristics of the study population (Mean values and standard deviations; median values and interquartile ranges; numbers and percentages)

	Total (*n* 53)		Omnivore (*n* 18)		Vegetarian (*n* 21)		Vegan (*n* 14)		
	Mean	sd	Mean	sd	Mean	sd	Mean	sd	*P*
Age (years)	29·8	5·9	30·2	5·9	29·3	5·8	29·9	6·2	0·89
Male									
* n*	24		12		7		5		0·08
%	45·3		66·7		33·3		35·7		
Daily average calories (cal)									
Median	1468		1515		1450		1405		0·94
IQR	1120–1790		1150–1790		1230–1780		1250–1770		
Daily protein intake (g)	50·9	10·4	55·1	8·2	46·9	10·6	51·8	10·9	**0·04**
Daily steps, *1000	13·6	3·3	13·1	3·3	14·0	3·1	13·6	3·7	0·71
BMI (kg/m^2^)	24·8	3·4	25·2	3·5	24·5	3·6	24·6	3·2	0·82
Fat mass (kg)	18·6	6·5	18·9	5·8	18·9	7·9	16·7	5·3	0·84
Fat percentage (%)	25·6	6·9	25·4·	6·9	26·1·	8·3	25·2·	4·4	0·92
Obesity degree (%)	10·0	14·8	11·4	14·5	9·6	16·7	8·9	12·9	0·88
Fat free mass (kg)	53·8	11·3	56·9	11·1	52·3	11·3	52·0	11·3	0·36
Muscle mass (kg)	51·8	10·7	54·1·	10·6	51·4·	10·8	49·4·	10·8	0·47
SBP (mmHg)	117·6	10·3	121·7	8·4	114·8	10·3	116·8	11·5	0·11
DBP (mmHg)	73·9	6·5	76·1	5·8	73·6	5·0	71·8	8·5	0·16
	Median	IQR	Median	IQR	Median	IQR	Median	IQR	
Total hilus	2·8	1·9–4·8	2·3	1·9–3·7	3·0	1·8–6·1	3·1	2·7–5·4	0·28
Liver 2 (%)	2·0	1·7–3·5	2·1	1·8–3·5	1·8	1·6–5·4	2·1	1·5–3·3	0·69
Liver 4b (%)	1·9	1·6–4·4	1·9	1·6–3·8	1·8	1·4–5·8	2·0	1·4–3·3	0·98
Liver 4a (%)	2·0	1·6–3·4	2·1	1·7–3·0	1·8	1·6–4·6	1·9	1·5–3·3	0·81
Liver 8 (%)	2·3	1·8–4·6	2·5	1·9–4·3	2·3	1·8–4·9	2·2	1·5–3·8	0·82
Liver 7 (%)	2·2	1·6–3·7	2·3	1·9–2·5	2·1	1·6–4·4	1·9	1·2–3·7	0·62
Liver 3 (%)	2·0	1·6–3·6	2·1	1·9–3·2	1·7	1·5–3·9	1·8	1·5–3·9	0·66
Liver 5 (%)	2·1	1·5–3·9	2·1	1·8–2·9	2·0	1·4–5·2	2·2	1·4–3·8	0·94
Liver 6 (%)	2·2	1·7–3·6	2·3	1·9–2·8	2·0	1·7–6·8	2·4	1·3–3·6	0·85
Liver 1 (%)	2·2	1·7–4·0	2·2	1·8–3·1	1·9	1·6–4·7	2·2	1·5–4·0	0·91
Kidney upper (%)	1·6	1·3–2·0	1·7	1·3–2·4	1·5	1·3–1·8	1·7	1·4–2·2	0·37
Kidney mid (%)	1·6	1·4–1·9	1·6	1·3–2·2	1·5	1·4–1·9	1·8	1·5–1·9	0·59
Kidney lower (%)	1·6	1·4–2·0	1·6	1·4–1·7	1·5	1·3–1·8	1·9	1·5–2·1	0·25
Kidney total (%)	1·5	1·3–2·0	1·9	1·5–2·2	1·4	1·2–1·8	1·6	1·3–1·9	0·14

DBP, diastolic blood pressure; SBP, systolic blood pressure.

*P*, the *P*-value for each characteristic represents an overall comparison across omnivorous, vegetarian and vegan groups.

Clinical parameters evaluated in our study included BP measurements, BMI assessment, and body fat and muscle mass assessment via the Tanita MC-780 S Black Segmental Body Composition Analyzer. This device uses bioelectrical impedance analysis to assess body composition. Biochemical parameters investigated in this study included the complete blood count, serum levels of alanine aminotransferase, aspartate aminotransferase, alkaline phosphatase, direct bilirubin, high sensitive C-reactive protein, creatinine, blood urea nitrogen, fasting glucose and insulin, Homeostatic Model Assessment for Insulin Resistance (HOMA-IR), uric acid, total cholesterol, LDL-cholesterol, HDL-cholesterol, TAG and urinary albumin-to-creatinine ratio (Microalbuminuria). Those clinical and biochemical parameters were evaluated at baseline and 6-month follow-up visits.

Liver and kidney steatosis were evaluated at baseline and at the 6-month follow-up using MRI with the proton density fat fraction (PDFF) technique, which estimates fat content in tissues. The MRI-PDFF technique is one of the most accurate non-invasive methods for assessing steatosis, with a strong correlation to histopathological findings and the ability to quantify steatosis^(24,25)^.

### Statistical analysis

Data are presented as mean (standard deviation), median with interquartile range or number and percent frequency, as appropriate. The comparison between groups was performed using the *χ* ^2^ test for categorical variables and the Kruskal–Wallis, one-way ANOVA, Mann–Whitney or independent *t* test for the remaining variables, as appropriate. The normality of the distribution was assessed using the Shapiro–Wilk test.

Time-repeated measurements were analysed using linear mixed models including group, time and the group-by-time interaction term. Normally distributed continuous variables were assessed through mixed models for repeated measurements, and for non-normally distributed data, penalised quasi-likelihood under restricted maximum likelihood models was applied. All models were adjusted for baseline values, daily average energy content, daily average protein intake and daily step counts at baseline and 6 months. A *P*-value of less than 0·05 was considered statistically significant. Power analysis was performed to determine the minimum number of participants needed for the study, as described previously. All analyses were conducted using Stata MP Software, version 13 (Stata Statistical Software: Release 13. StataCorp LP).

## Results

### Baseline characteristics

The total number of participants who completed the study was 53, divided into 18, 21 and 14 participants in the omnivorous, vegetarian and vegan groups, respectively, with differences due to participant dropout throughout the study period (Fig. 1). Baseline demographic, clinical, imaging and biochemical characteristics are presented in Table 1. An overall comparison across the three groups indicated a significant difference in daily protein intake (*P* = 0·04), with the omnivorous group having a higher mean protein intake compared with the vegetarian group. There were no other significant differences between the three groups.

### Changes in anthropometric and clinical parameters during follow-up

**Fig. 2.**
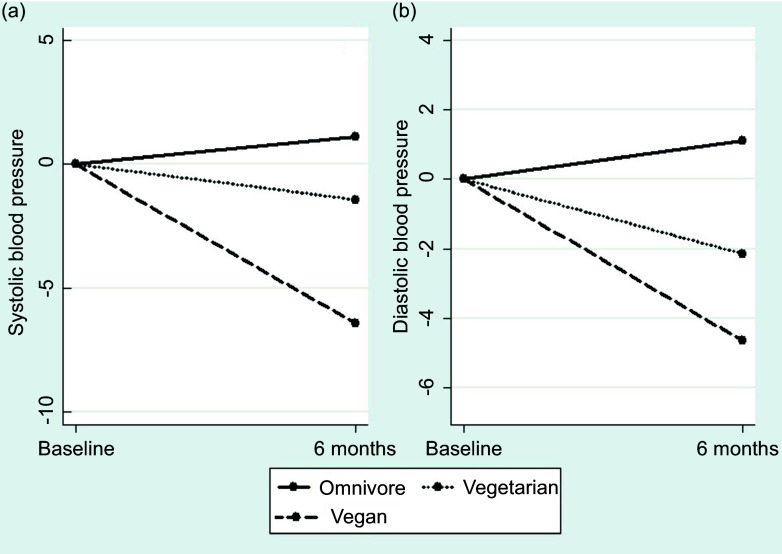
Systolic and diastolic blood pressures evolution during the follow-up across the three groups.

First, we assessed the changes in BMI, body compartments, systolic BP and diastolic BP at the 6-month follow-up (see Table 2). No significant changes in BMI, fat mass, fat percentage or muscle mass were observed between the three groups during the follow-up period. However, a significant difference in the slope of fat-free mass change was observed between the three groups (0·97, 95 % CI 0·19, 1·75, –0·03, 95 % CI –0·76, 0·69 and –0·89, 95 % CI –1·78, –0·01 for the omnivorous, vegetarian and vegan groups, respectively). Similar results were observed for fat mass. By contrast, systolic and diastolic BP decreased significantly at 6 months, with a significant difference in the slope of decrease between the three groups (Fig. 2). Additionally, there was no statistically significant change in daily step counts between baseline and 6 months across the groups.

**Table 2.** BMI, body compartments and systolic and diastolic blood pressure evolution during the follow-up across the three groups (Mean values and 95 % confidence intervals)

	Baseline		6 months			
	Mean	95 % CI	Mean	95 % CI	*P* ^*^	*P* ^†^
BMI (kg/m^2^)						
Omnivore (*n* 18)	25·2	23·6, 26·8	25·8	24·2, 27·4	0·49	0·52
Vegetarian (*n* 21)	24·5	23·0, 26·0	24·8	23·3, 26·3		
Vegan (*n* 14)	24·6	22·8, 26·4	24·8	22·9, 26·6		
* P* ^‡^	–		0·61			
Fat mass						
Omnivore (*n* 18)	18·9	16·1, 21·8	19·9	16·9, 22·7	0·58	**0·02**
Vegetarian (*n* 21)	18·9	16·2, 21·5	19·4	16·7, 22·0		
Vegan (*n* 14)	17·7	14·4, 20·9	17·1	13·8, 20·3		
* P* ^‡^			**0·002**			
Fat percentage						
Omnivore (*n* 18)	25·4	22·5, 28·4	25·7	22·7, 28·7	0·25	0·51
Vegetarian (*n* 21)	26·1	23·3, 28·8	26·6	23·9, 29·3		
Vegan (*n* 14)	25·2	21·8, 28·5	24·9	21·6, 28·3		
* P* ^‡^			0·37			
Fat free mass						
Omnivore (*n* 18)	56·9	51·9, 61·9	57·9	52·9, 62·9	0·32	**0·004**
Vegetarian(*n* 21)	52·3	47·7, 56·9	52·3	47·6, 56·9		
Vegan (*n* 14)	52·1	46·4, 57·8	51·2	45·5, 56·9		
* P* ^‡^	–		**< 0·001**			
Muscle mass						
Omnivore (*n* 18)	54·1	49·1, 59·1	53·2	48·2, 58·3	**0·01**	0·61
Vegetarian (*n* 21)	51·4	46·8, 56·1	49·6	44·9, 54·3		
Vegan (*n* 14)	49·5	43·8, 55·1	48·7	43·1, 54·4		
* P* ^‡^			0·81			
SBP (mmHg)						
Omnivore (*n* 18)	121·7	117·4, 125·9	122·8	118·5, 127·1	**0·04**	**0·01**
Vegetarian (*n* 21)	114·8	110·8, 118·7	113·3	109·3, 117·3		
Vegan (*n* 14)	116·8	111·9, 121·7	110·4	105·5, 115·2		
* P* ^‡^	–		**< 0·001**			
DBP (mmHg)						
Omnivore (*n* 18)	76·1	73·4, 78·8	77·2	74·5, 79·9	**0·002**	**0·004**
Vegetarian (*n* 21)	73·6	71·1, 76·1	71·4	68·9, 73·9		
Vegan (*n* 14)	71·8	68·7, 74·8	67·1	64·1, 70·2		
* P* ^‡^	–		**< 0·001**			

DBP, diastolic blood pressure; SBP, systolic blood pressure.

Data are presented as mean (95 % CI) at baseline, and least-squares mean (95 % CI) at 6 months. Analysis was conducted using a mixed model for repeated measures, adjusting for baseline values and for baseline and 6 months daily average calories, daily average proteins and daily steps.

* *P* value for time effect – trend over time in all arms.

† *P* value for treatment × time interaction – evaluates if changes in one group are different from the changes in other groups.

‡ *P* value for comparison between groups at each moment.

Bold values represent statistically significant.

### Changes in biochemical parameters during follow-up

We have analysed the evolution of different biochemical analyses during the 6 months of intervention. No significant differences were observed in liver function tests (aspartate aminotransferase, alanine aminotransferase, or total and direct bilirubin) or Hb levels (Table 3). However, a significant difference in the slope of change for serum glucose and insulin was observed, though no significant changes in HOMA-IR were noted.

**Table 3.** Biological parameters, liver and kidney PDFF values evolution during the follow-up across the three groups (Mean values and 95 % confidence intervals)

	Baseline		6 months			
	Mean	95 % CI	Mean	95 % CI	*P* ^*^	*P* ^†^
Serum glucose (mg/dl)						
Omnivore (*n* 18)	90·4	87·2, 93·6	94·5	91·3, 97·7	0·71	**0·006**
Vegetarian (*n* 21)	91·9	88·9, 94·8	91·6	88·7, 94·6		
Vegan (*n* 14)	91·8	88·2, 95·4	90·2	86·6, 93·8		
* P* ^‡^	–		**0·001**			
Insulin (µU/ml)						
Omnivore (*n* 18)	10·5	5·8, 15·2	16·8	12·0, 21·5	0·71	**0·006**
Vegetarian (*n* 21)	10·5	6·1, 14·9	8·7	4·3, 13·1		
Vegan (*n* 14)	8·9	3·5, 14·3	6·9	1·5, 12·3		
* P* ^‡^	–		**0·001**			
HOMA score						
Omnivore (*n* 18)	2·4	1·3, 3·4	3·3	2·2, 4·3	0·59	0·13
Vegetarian (*n* 21)	2·4	1·5, 3·4	2·4	1·4, 3·4		
Vegan (*n* 14)	2·1	0·9, 3·3	1·5	0·3, 2·7		
* P* ^‡^	–		0·10			
Total cholesterol (mg/dl)						
Omnivore (*n* 18)	178·4	162·4, 194·4	175·8	159·8, 191·8	0·18	0·13
Vegetarian (*n* 21)	171·2	156·4, 186·1	169·4	154·6, 184·2		
Vegan (*n* 14)	178·4	160·2, 196·5	160·5	142·4, 178·6		
* P* ^‡^	–		**0·01**			
HDL-cholesterol (mg/dl)						
Omnivore (*n* 18)	54·7	49·1, 60·4	56·5	50·9, 62·1	0·19	**0·04**
Vegetarian (*n* 21)	63·0	57·8, 68·3	60·4	55·2, 65·6		
Vegan (*n* 14)	60·2	53·8, 66·6	55·4	48·9, 61·7		
* P* ^‡^	–		**0·001**			
LDL-cholesterol (mg/dl)						
Omnivore (*n* 18)	117·9	102·9, 132·8	117·4	102·4, 132·4	**0·04**	**0·006**
Vegetarian (*n* 21)	105·8	92·0, 119·6	104·0	90·3, 117·8		
Vegan (*n* 14)	119·1	102·2, 136·0	101·4	84·5, 118·3		
* P* ^‡^	–		**< 0·001**			
TAG (mg/dl)						
Omnivore (*n* 18)	79·6	58·9, 100·3	75·4	54·4, 96·4	0·08	0·91
Vegetarian (*n* 21)	75·4	54·4, 96·4	87·3	68·2, 106·5		
Vegan (*n* 14)	97·2	73·8, 120·7	86·1	62·7, 109·6		
* P* ^‡^	–		0·82			
Liver, total hilus (%)						
Omnivore (*n* 18)	2·9	1·6, 4·2	4·2	2·9, 5·5	0·47	**0·04**
Vegetarian (*n* 21)	3·9	2·7, 5·1	3·8	2·6, 5·0		
Vegan (*n* 14)	2·9	1·4, 4·4	2·5	1·0, 3·9		
* P* ^‡^	–		**0·003**			
Liver Segment 6 (%)						
Omnivore (*n* 18)	79·6	58·9, 100·3	75·4	54·4, 96·4	0·08	0·91
Vegetarian (*n* 21)	75·4	54·4, 96·4	87·3	68·2, 106·5		
Vegan (*n* 14)	97·2	73·8, 120·7	86·1	62·7, 109·6		
* P* ^‡^	–		0·82			
Kidney total (%)						
Omnivore (*n* 18)	1·9	1·6, 2·2	2·0	1·7, 2·3	0·56	0·88
Vegetarian (*n* 21)	1·7	1·4, 1·9	1·8	1·5, 2·0		
Vegan (*n* 14)	1·7	1·4, 2.	1·8	1·5, 2·1		
* P* ^‡^	–		0·70			
Kidney lower (%)						
Omnivore (*n* 18)	1·7	1·5, 1·9	1·9	1·8, 2·2	0·28	**< 0·001**
Vegetarian (*n* 21)	1·7	1·4, 1·9	1·7	1·5, 1·9		
Vegan (*n* 14)	1·9	1·6, 2·1	1·4	1·2, 1·7		
* P* ^‡^	–		**< 0·001**			
eGFR, ml/min/1·73 m^2^						
Omnivore (*n* 18)	118·3	110·9, 125·7	107·9	100·5, 115·4	**< 0·001**	0·02
Vegetarian (*n* 21)	118·4	111·6, 125·3	118·5	111·6, 125·3		
Vegan (*n* 14)	125·6	117·2, 134·1	118·9	110·4, 127·3		
* P* ^‡^	–		**0·002**			

PDFF, Proton Density Fat Fraction; HOMA, Homeostatic Model Assessment for Insulin Resistance.

Data are presented as mean (95 % CI) at baseline, and least-squares mean (95 % CI) at 6 months. Analysis was conducted using a mixed model for repeated measures, adjusting for baseline values and for baseline and 6 months daily average calories, daily average proteins and daily steps.

* *P* value for time effect – trend over time in all arms.

† *P* value for treatment × time interaction – evaluates if changes in one group are different from the changes in other groups.

‡ *P* value for comparison between groups at each moment.

Bold values represent statistically significant.

Regarding the lipid levels, there were no significant changes in total cholesterol values. However, HDL- and LDL-cholesterol levels decreased more significantly in the vegan group during the follow-up. Additionally, there was a significant decrease in estimated glomerular filtration rate (eGFR) levels, with a significant difference in the slope between the three groups (Table 3).

### Changes at liver and kidney steatosis on imaging during follow-up

**Fig. 3.**
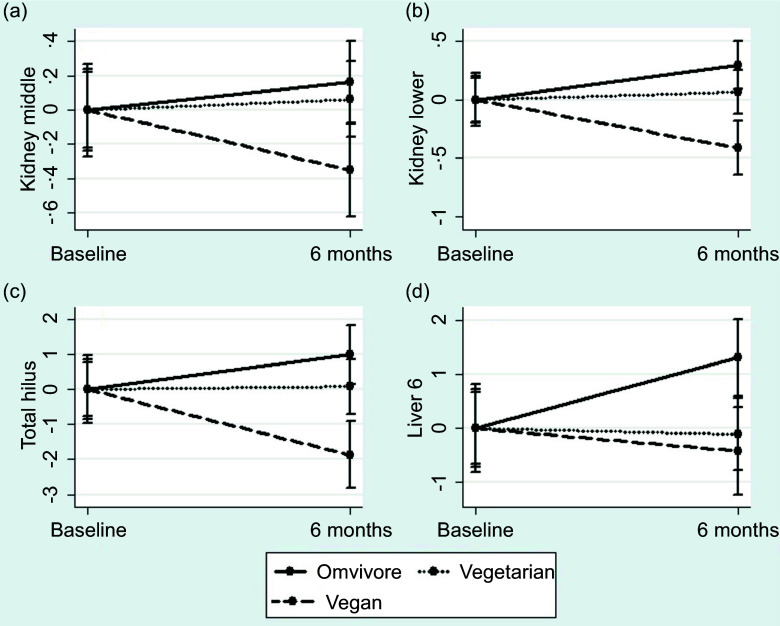
Kidney and liver MRI-proton density fat fraction value evolution during the follow-up across the three groups.

Lastly, a significant difference in the slope of change in kidney steatosis at the lower pole was identified between the three groups (Fig. 3(a) and (b)). No other significant differences in kidney parameters were observed. For liver parameters, significant differences in the slope of change were observed for the total hilus (*P* = 0·008) and the Liver 6 indexes (*P* = 0·04) (Fig. 3(c) and (d)).

## Discussion

We performed a single-centre prospective study in fifty-three participants, to investigate the effects of three major dietary patterns on various anthropometric and biochemical parameters, as well as liver and kidney steatosis, assessed using the MRI-PDFF technique at the 6-month follow-up. Analysis of the results showed no significant changes in BMI, fat mass, fat percentage or muscle mass between the three dietary groups.

Our findings indicated that the vegan diet was associated with statistically significant improvements in liver steatosis, particularly at segment 6 of the liver and reductions in kidney steatosis at the lower pole. Additionally, participants on a vegan diet showed decreases in LDL- and HDL-cholesterol and improvements in both systolic and diastolic BP. In contrast, no significant changes were observed in the vegetarian group. This lack of change in the vegetarian group may be partly attributable to the inclusion of dairy products and eggs, which contain saturated fats and cholesterol that could attenuate improvements in metabolic parameters. Saturated fats have been linked to hepatic fat accumulation, and dietary cholesterol may influence lipid levels, potentially contributing to less favourable effects on liver and cardiovascular outcomes compared with a fully plant-based diet^(26)^. Further, the statistically significant increase in eGFR observed in the vegan and vegetarian groups may be partly due to improvements in ‘fatty kidney’ status, as reduced fat deposits in renal tissues have been linked to better kidney function^(27)^. Additionally, the lower acid load associated with plant-based diets may have contributed to these findings, as a reduced dietary acid load can lessen renal acid excretion demands, potentially preserving kidney function^(28,29)^.

Dietary and lifestyle modifications are the cornerstone of both the preventive and therapeutic approach towards NAFLD; however, the optimal dietary modifications remain under investigation. Vegetarian and vegan diets, which emphasise whole grains, vegetables, fruits, legumes and nuts, have shown promising benefits for liver health, with several studies associating these diets with reductions in hepatic steatosis, improved liver enzyme levels and better metabolic outcomes in patients with NAFLD^(30–32)^. These plant-based diets are low in saturated fats and dietary cholesterol, which may reduce fat accumulation in the liver and improve lipid profiles, potentially contributing to a lower risk of insulin resistance and inflammation^(33)^. Studies have also suggested that plant-based diets may lower oxidative stress, support favourable changes in the gut microbiome and improve glucose metabolism, further aiding in NAFLD management^(31,34)^. While direct comparisons between vegetarian, vegan and other diets in NAFLD are limited, the unique nutrient composition of plant-based diets – high in fibre, antioxidants, and phytochemicals – may offer a protective effect on liver function.

To the best of our knowledge, our prospective study is the first study investigating the role of various dietary modalities on renal steatosis. Our prospective study is among the few to examine the effects of vegetarian and vegan diets on hepatic and renal steatosis using MRI-PDFF, a non-invasive assessment method^(35)^. Although some data are available on dietary patterns for liver health, data on renal steatosis remain sparse^(36)^. Recent research suggests that increased renal steatosis is linked to higher risks of chronic kidney disease and cardiovascular events, underscoring the clinical relevance of our study^(34)^.

A recent open-label prospective study including forty patients with NAFLD demonstrated significant improvements in liver enzymes, with alanine aminotransferase decreasing from 99 U/L (sd 45) to 36 U/L (sd 21) and aspartate aminotransferase from 54 U/L (sd 44) to 27 U/L (sd 10) after 6 months on a strict vegan diet^(37)^. However, this study was limited by the lack of a control group, a high dropout rate (fourteen patients, 35 %), and no evaluation of hepatosteatosis. In contrast, our study included participants without liver disease at baseline, so alanine aminotransferase and aspartate aminotransferase levels were already within the normal range, resulting in only modest reductions among vegan participants. Our study’s dropout rate in the vegan group was similar at 30 %, but our inclusion of a control group allowed for broader comparisons across dietary interventions. A randomised controlled trial of 244 participants, using proton magnetic resonance spectroscopy to assess lipids, assigned participants to a low-fat vegan or regular diet for 16 weeks and found significant improvements in body weight (−5·9 kg; *P*< 0·001), insulin sensitivity (HOMA-IR − 1·3; *P*< 0·001) and reductions in hepatocellular (−34·4 %; *P* = 0·002) and intramyocellular lipids (−10·4 %; *P* = 0·03) in the vegan group^(15)^. Our study, conducted over 6 months in participants without baseline liver disease, found modest reductions in liver fat due to already-normal HOMA-IR and hepatic lipid levels at baseline. While both studies highlight metabolic benefits of a vegan diet, ours uniquely assessed renal steatosis using MRI-PDFF.

Even though the exact biochemical and pathophysiological mechanisms leading to difference in terms of various dietary habits are largely unknown, there are multiple hypothesis in this regard. Plant-based diets typically contain higher proportions of unsaturated fatty acids and are rich in dietary fibre and phytosterols, which have been shown to reduce LDL-cholesterol and improve lipid profiles, reducing the lipid accumulation that contributes to liver steatosis^(38,39)^. Additionally, plant-based fats, dietary fibre and phytochemicals exhibit anti-inflammatory effects that modulate gut microbiota composition, potentially reducing systemic inflammation – a factor implicated in both liver and kidney disease progression^(40–43)^. A lower salt load in plant-based diets has been shown to help maintain kidney function by reducing hypertension, while the absence of heme iron may reduce oxidative stress, a known contributor to liver and kidney damage^(44,45)^.

Our prospective study has several important considerations limiting the generalisability of our results. First, the 6-month follow-up period may have limited our ability to detect long-term changes in outcomes such as liver and kidney steatosis, as well as sustained alterations in anthropometric and biochemical parameters. Second, although MRI-PDFF is highly reliable, histopathological assessment remains the gold standard for evaluating liver and kidney steatosis^(46,47)^. Additionally, the lack of dietary compliance assessment remains a significant limitation in our study. Participants self-selected into each diet group, potentially introducing variability in adherence due to differing health motivations and perceptions. Participants choosing to maintain their usual omnivorous diet may have been less inclined to modify their dietary habits, possibly reflecting fewer immediate health concerns or a preference for continuity rather than dietary change. In contrast, those selecting vegetarian or vegan diets may have been motivated by a desire to improve health outcomes, potentially related to unreported or subclinical health issues. This variability in motivation could have influenced adherence levels across groups, as participants actively seeking dietary change may demonstrate greater compliance with the intervention. Future studies could address this by employing randomised group assignments or standardised adherence monitoring to better evaluate the effects of dietary interventions. Moreover, a detailed assessment of dietary compliance represents a limitation in this study. Although monthly nutritional diaries were collected and examined, it was possible to evaluate only general dietary patterns rather than precise quantities and specific nutritional content. Consequently, an accurate assessment of macronutrient and micronutrient intake across omnivorous, vegetarian and vegan groups was not feasible, limiting the capacity to evaluate adherence comprehensively. Moreover, although there is a male predominance among omnivore group participants (66·7 %) compared with vegetarian (33·3 %) and vegan group (35·7 %), we have adjusted for this relative imbalance in our analysis. Also, our analysis lack data regarding the daily micro- and macronutrient intake of participants from different groups as well as lack of standardised method to assess the dietary adherence except from interviews and 3-d food intake logbooks. Lastly, the statistically higher daily energetic intake may potentially contribute to the differences regarding the adipose tissue measurements on MRI scans. Nevertheless, the results of our prospective study are significant and could potentially enlighten future clinical trials that would investigate the effects of dietary habits on multiple clinical parameters and end points.

## Supporting information

Guler Senturk et al. supplementary material 1 Guler Senturk et al. supplementary material

Guler Senturk et al. supplementary material 2 Guler Senturk et al. supplementary material

## Supplementary material

For supplementary material/s referred to in this article, please visit https://doi.org/10.1017/S0007114525000017
