# Supplementary material for: Effects of plant-based diet on metabolic parameters, liver and kidney steatosis: a prospective interventional open-label study
Source: Br J Nutr. 2025 Jan 10;133(3):289–98. doi: 10.1017/S0007114525000017 (PMC11946044; doi:10.1017/S0007114525000017)
Supplement: Guler Senturk et al. supplementary material 1 — Guler Senturk et al. supplementary material [file S0007114525000017sup001.docx]

**Supplementary Table 1.** Kidney magnetic resonance imaging-proton density fat fraction values evolution during the follow-up across the three groups.

|  | **Baseline, (%)** | **6 Months, (%)** | **p*** | **p^†^** |
| --- | --- | --- | --- | --- |
| **Kidney upper, (%)** |  |  |  |  |
| Omnivore (N=18) | 1.9 (1.6-2.2) | 1.8 (1.5-2.1) | 0.06 | 0.17 |
| Vegetarian (N=21) | 1.7 (1.4-1.9) | 1.8 (1.5-2.0) |  |  |
| Vegan (N=14) | 1.9 (1.6-2.3) | 1.6 (1.3-1.9) |  |  |
| p^‡^ | - | 0.16 |  |  |
| **Kidney middle, (%)** |  |  |  |  |
| Omnivore (N=18) | 1.8 (1.5-2.1) | 1.9 (1.7-2.2) | 0.25 | 0.09 |
| Vegetarian (N=21) | 1.7 (1.4-1.9) | 1.8 (1.5-2.0) |  |  |
| Vegan (N=14) | 1.9 (1.6-2.2) | 1.6 (1.3-1.9) |  |  |
| p^‡^ | - | **0.02** |  |  |
| **Kidney lower, (%)** |  |  |  |  |
| Omnivore (N=18) | 1.7 (1.5-1.9) | 1.9 (1.8-2.2) | 0.28 | **<0.001** |
| Vegetarian (N=21) | 1.7 (1.4-1.9) | 1.7 (1.5-1.9) |  |  |
| Vegan (N=14) | 1.9 (1.6-2.1) | 1.4 (1.2-1.7) |  |  |
| p^‡^ | - | **<0.001** |  |  |
| **Kidney total, (%)** |  |  |  |  |
| Omnivore (N=18) | 1.9 (1.6-2.2) | 2.0 (1.7-2.3) | 0.56 | 0.88 |
| Vegetarian (N=21) | 1.7 (1.4-1.9) | 1.8 (1.5-2.0) |  |  |
| Vegan (N=14) | 1.7 (1.4-2.) | 1.8 (1.5-2.1) |  |  |
| p^‡^ | - | 0.70 |  |  |

Data are presented as mean (95%CI) at baseline, and least-squares mean (95%CI) at 6 months. Analysis was conducted using a mixed model for repeated measures, adjusting for baseline values and for baseline and 6 months daily average calories, daily average proteins and daily steps.

*P value for time effect – trend over time in all arms

^†^P value for treatment x time interaction – evaluates if changes in one group are different from the changes in other groups

^‡^P value for comparison between groups at each moment
