# Supplementary material for: Effects of plant-based diet on metabolic parameters, liver and kidney steatosis: a prospective interventional open-label study
Source: Br J Nutr. 2025 Jan 10;133(3):289–98. doi: 10.1017/S0007114525000017 (PMC11946044; doi:10.1017/S0007114525000017)
Supplement: Guler Senturk et al. supplementary material 2 — Guler Senturk et al. supplementary material [file S0007114525000017sup002.docx]

**Supplementary Table 2.** Liver magnetic resonance imaging-proton density fat fraction values evolution during the follow-up across the three groups.

|  | Baseline, (%) | 6 Months, (%) | p* | p^†^ |
| --- | --- | --- | --- | --- |
| **Total hilus, (%)** |  |  |  |  |
| Omnivore (N=18) | 3.3 (1.7-4.9) | 4.3 (2.7-5.8) | 0.28 | **0.008** |
| Vegetarian (N=21) | 4.5 (3.1-5.9) | 4.6 (3.1-6.0) |  |  |
| Vegan (N=14) | 4.4 (2.6-6.1) | 2.5 (0.7-4.3) |  |  |
| p^‡^ | - | **<0.001** |  |  |
| **Liver 2, (%)** |  |  |  |  |
| Omnivore (N=18) | 3.1 (1.8-4.4) | 3.7 (2.4-4.9) | 0.77 | 0.36 |
| Vegetarian (N=21) | 3.6 (2.4-4.8) | 3.3 (2.1-4.5) |  |  |
| Vegan (N=14) | 2.8 (1.3-4.2) | 2.2 (0.7-3.7) |  |  |
| p^‡^ | - | 0.08 |  |  |
| **Liver 4b, (%)** |  |  |  |  |
| Omnivore (N=18) | 2.9 (1.6-4.3) | 3.9 (2.5-5.2) | 0.76 | 0.19 |
| Vegetarian (N=21) | 3.7 (2.4-4.9) | 3.5 (2.3-4.8) |  |  |
| Vegan (N=14) | 2.9 (1.4-4.4) | 2.6 (1.1-4.1) |  |  |
| p^‡^ | - | 0.05 |  |  |
| **Liver 4a, (%)** |  |  |  |  |
| Omnivore (N=18) | 2.9 (1.6-4.1) | 3.5 (2.3-4.8) | 0.30 | 0.39 |
| Vegetarian (N=21) | 3.6 (2.4-4.8) | 3.8 (2.6-4.9) |  |  |
| Vegan (N=14) | 2.8 (1.4-4.2) | 2.5 (1.0-3.9) |  |  |
| p^‡^ | - | 0.08 |  |  |
| **Liver 8, (%)** |  |  |  |  |
| Omnivore (N=18) | 3.5 (1.9-5.1) | 4.4 (2.8-5.9) | 0.57 | 0.29 |
| Vegetarian (N=21) | 4.2 (2.7-5.7) | 4.8 (3.3-6.3) |  |  |
| Vegan (N=14) | 3.2 (1.4-5.0) | 2.7 (0.8-4.5) |  |  |
| p^‡^ |  | 0.08 |  |  |
| **Liver 7, (%)** |  |  |  |  |
| Omnivore (N=18) | 3.2 (1.5-4.8) | 4.2 (2.6-5.9) | 0.43 | 0.33 |
| Vegetarian (N=21) | 3.6 (2.1-5.2) | 4.8 (3.2-6.3) |  |  |
| Vegan (N=14) | 3.0 (1.1-4.9) | 2.6 (0.8-4.5) |  |  |
| p^‡^ | - | 0.12 |  |  |
| **Liver 3, (%)** |  |  |  |  |
| Omnivore (N=18) | 2.9 (1.6-4.2) | 3.8 (2.5-5.1) | 0.83 | 0.16 |
| Vegetarian (N=21) | 3.6 (2.3-4.8) | 3.4 (2.2-4.6) |  |  |
| Vegan (N=14) | 3.0 (1.5-4.5) | 2.2 (0.7-3.7) |  |  |
| p^‡^ | - | **0.01** |  |  |
| **Liver 5, (%)** |  |  |  |  |
| Omnivore (N=18) | 2.9 (1.4-4.4) | 4.0 (2.6-5.5) | 0.06 | 0.12 |
| Vegetarian (N=21) | 3.9 (2.5-5.3) | 4.9 (3.5-6.3) |  |  |
| Vegan (N=14) | 2.9 (1.3-4.6) | 2.7 (1.1-4.4) |  |  |
| p^‡^ | - | **0.03** |  |  |
| **Liver 6, (%)** |  |  |  |  |
| Omnivore (N=18) | 2.9 (1.6-4.2) | 4.2 (2.9-5.5) | 0.47 | **0.04** |
| Vegetarian (N=21) | 3.9 (2.7-5.1) | 3.8 (2.6-5.0) |  |  |
| Vegan (N=14) | 2.9 (1.4-4.4) | 2.5 (1.0-3.9) |  |  |
| p^‡^ | - | **0.003** |  |  |
| **Liver 1, (%)** |  |  |  |  |
| Omnivore (N=18) | 3.0 (1.9-4.1) | 3.4 (2.4-4.5) | 0.37 | 0.22 |
| Vegetarian (N=21) | 3.2 (2.2-4.2) | 3.3 (2.3-4.3) |  |  |
| Vegan (N=14) | 2.9 (1.8-4.2) | 2.1 (0.9-3.3) |  |  |
| p^‡^ | - | **0.03** |  |  |

Data are presented as mean (95%CI) at baseline, and least-squares mean (95%CI) at 6 months. Analysis was conducted using a mixed model for repeated measures, adjusting for baseline values and for baseline and 6 months daily average calories, daily average proteins and daily steps.

*P value for time effect – trend over time in all arms

^†^P value for treatment x time interaction – evaluates if changes in one group are different from the changes in other groups

^‡^P value for comparison between groups at each moment
